# On-Scene Emergency Department at Aalborg carnival

**DOI:** 10.1186/1757-7241-20-S2-P34

**Published:** 2012-04-16

**Authors:** Lars-Gustav Rahbek Nielsen, Michael Kanstrup Dahl

**Affiliations:** 1 Aalborg Hospital, Aarhus University Hospital, Department of Anaesthesiology, North Denmark Region, Denmark

## Background

Every year in May, the biggest carnival in Scandinavia takes place in Aalborg. 100.000 people are gathered in the streets to celebrate, and of these more than 25.000 enters the carnival compound. During the day, the streets of central Aalborg are effectively closed due to the massive number of people.

Historically, Danish Red Cross volunteers have set up first aid stations and treatment facilities during the carnival, staffed with first aid trained personnel to care for injuries. They would refer more serious patients to the Emergency Department, Aalborg Hospital (EDAH).

We describe our experience with an On-Scene Emergency Department (OED) staffed with senior doctors and nurses during the 2010 and 2011 Aalborg Carnival in terms of injury types, level of treatment, reducing patient load on the EDAH, and economics.

## Methods

All patient contacts for the years 2010 -2011 were recorded on a custom-made medical chart. Data is entered into a Microsoft Access 2007 database and processed in Microsoft Excel 2007. The database has been approved by the Danish Data Protection Agency.

The study did not involve any experimental procedures.

## Results

A total of 456 carnival-related patients were consulted in the OED and the EDAH in 2010 and 2011.

53% (n=240) were consulted in OED. 17% (n=41) of patients needed hospitalization, primary for x-ray, and were admitted to the EDAH. 83% (n=199) was finalized and returning to the carnival or home. 54% (n=131) had wounds and lacerations.

The Diagnosis-Related Group (DRG) value of the treatments performed in OED is DKR 199.000.

## Conclusion

Placing an OED, staffed with senior doctors, at major cultural events with many participants, is a viable option, reducing load on conventional Emergency Departments, providing good level of care and good service to the participants.

The DRG value seems sufficient to cover the salary expenses of doctors and nurses present.

